# Periprosthetic joint infections in modular endoprostheses of the lower extremities: a retrospective observational study in 101 patients

**DOI:** 10.1186/s13037-016-0095-8

**Published:** 2016-02-09

**Authors:** Dirk Zajonz, Almut Zieme, Torsten Prietzel, Michael Moche, Solveig Tiepoldt, Andreas Roth, Christoph Josten, Georg Freiherr von Salis-Soglio, Christoph- E. Heyde, Mohamed Ghanem

**Affiliations:** Department of Orthopaedic Surgery, Traumatology and Plastic Surgery, University Hospital Leipzig, Liebigstrasse 20, 04103 Leipzig, Germany; Department of Diagnostic and Interventional Radiology, University Hospital Leipzig, Liebigstrasse 20, 04103 Leipzig, Germany; Department of Nuclear medicine, University Hospital Leipzig, Liebigstrasse 20, 04103 Leipzig, Germany

**Keywords:** Periprosthetic infection, Endoprosthesis infection, Modular endoprosthesis, Mega-endoprosthesis

## Abstract

**Background:**

Modular mega-endoprosthesis systems are used to bridge very large bone defects and have become a widespread method in orthopaedic surgery for the treatment of tumours and revision arthroplasty. However, the indications for the use of modular mega-endoprostheses must be carefully considered. Implanting modular endoprostheses requires major, complication-prone surgery in which the limited salvage procedures should always be borne in mind. The management of periprosthetic infection is particularly difficult and beset with problems.

Given this, the present study was designed to gauge the significance of periprosthetic infections in connection with modular mega-implants in the lower extremities among our own patients.

**Methods:**

Patients who had been fitted with modular endoprosthesis on a lower extremity at our department between September 1994 and December 2011 were examined retrospectively. A total of 101 patients with 114 modular prostheses were identified. Comprising 30 men (29.7 %) and 71 women (70.3 %), their average age at the time of surgery was 67 years (18–92 years).

**Results:**

The average follow-up period was 27 months (5 months and 2 weeks to 14 years and 11 months) and the drop-out rate was about 8.8 %. Altogether, there were 19 (17.7 %) endoprosthesis infections: 3 early infections and 16 late or delayed infections. The pathogen spectrum was dominated by coagulase-negative staphylococci (36 %) and *Staphylococcus aureus* (16 %), including 26 % multi-resistant pathogens. Reinfection occurred in 37 % of cases of infection. Tumours were followed by significantly fewer infections than the other indications. Infections were twice as likely to occur after previous surgery.

**Conclusion:**

In our findings modular endoprostheses (18 %) are much more susceptible to infection than primary endoprostheses (0.5–2,5 %). Infection is the most common complication alongside the dislocation of proximal femur endoprostheses. Consistent, radical surgery is essential – although even with an adequate treatment strategy, the recurrence rate is very high. Unfortunately, the functional results are frequently unsatisfactory, with amputation often being the last resort. Therefore, the indication for implantation must be carefully considered and discussed in great detail, especially in the case of multimorbid patients with previous joint infections.

## Background

Ever since Austin Moore fitted the first large metallic implant in a patient with a giant cell tumour of the proximal femur back in 1940, ‘mega-endoprostheses’ have become an accepted way of bridging large defects of long bones [1–3]. Early implants, which were made individually in an expensive, time-consuming process and whose intraoperative inflexibility was limited, have now largely been replaced by modular endoprosthesis systems. Primarily designed for the treatment of tumours, these systems are now used as standard in orthopaedic surgery for both primary and revision arthroplasty. Although the majority of experience has been obtained in tumour surgery, in the meantime the indications for mega-endoprostheses have substantially widened and other major defects, especially in connection with the replacement of existing prostheses, can also be bridged more successfully than with one-piece special implants. In particular, modular mega-endoprostheses are an important option in the event of loose implants, periprosthetic fractures with extensive osseous substance defects, periprosthetic infections and pseudarthrosis (non-union) or dislocation [4, 5]. As a broad market has developed for the use of mega-endoprostheses in revision arthroplasty as a result, almost all orthopaedic implant manufacturers now offer modular and revision systems to treat extensive substance defects of the lower extremities which in some cases can also be combined with the manufacturers’ primary implants [6].

With primary arthroplasty on the rise, cases of revision arthroplasty are also set to increase [7]. Since modular mega-endoprosthesis systems can be modified intraoperatively, they enable solutions even in complex cases where previously only unsatisfactory resection arthroplasty or amputations were possible and are being increasingly employed in revision arthroplasty [8–10]. However, the indications for the use of modular mega-endoprostheses must be strictly verified [10]. Implanting modular endoprostheses requires major, complication-prone surgery in which the limited salvage procedures should always be borne in mind. Due to numerous differences from primary arthroplasty such as the implants’ larger surface area, greater access, the frequently longer duration of surgery, higher blood loss and patients who are often multi-morbid, fitting mega-endoprostheses is associated with higher complication rates and should only be carried out by medical centres with the appropriate expertise [4]. This is particularly so in cases of periprosthetic infection with a lying mega-endoprosthesis, a high percentage of which lead to ablative surgery or even death [11].

Given this, the present study was designed to gauge the significance of periprosthetic infections in connection with modular mega-implants in the lower extremities among our own patients. This will allow risk factors to be identified relating in particular to indications or the patient population as well as problems compared to primary periprosthetic infections. Another goal is to report on the assessment of implant infections in modular systems in the current medical literature.

## Methods

To select the patient cohort, all the patients were retrospectively identified who had been fitted with a modular endoprosthesis in the lower extremity at our hospital between September 1994 and December 2011. Patients’ data was collected based on their archived records and electronic files in IS-H SAP (Siemens AG Healthcare Sector, Erlangen, Germany) as well as radiological findings and images from SIENET MagicWeb/ACOM (Siemens AG Healthcare Sector, Erlangen, Germany).

From the patient population, a total of 101 patients with 114 implanted modular endoprostheses were identified.

**Fig. 1:**
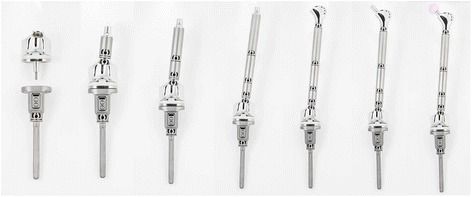
Different types of the modular system (By kind permission of AQ Implants, Ahrensburg, Germany)

The München-Lübeck™ modular endoprosthesis system (AQ Implants, Ahrensburg, Germany) was used. It was developed by Ascherl and Gradinger between 1992 and 1994, and first used in hospitals in 1994 [11, 12]. The system consists of extraosseous modules for the hip, knee and bone shaft as well as intramedullary rods. The individual components are attached by means of conical clamping and additional screw locking. Gradual length adjustment combined with the conical plug system allow the intraoperative selection and combination of individual implant components as well as rotation, antetorsion and curvature correction (Fig. 1) [10–12].

Statistical analysis was performed using the spreadsheet software Microsoft Excel (Microsoft Corporation, Redmond, USA). The *t*-test for two dependent samples was used to calculate significance, the level of significance being set at 5 % (α = 0.05).

The diagnosis of periprosthetic infection was provided on the basis of international consensus for the diagnosis of periprosthetic infection.

As reinfection or recurrence we defined a clinically and microbiologically recurrence of local periprosthetic joint infections according antibiotic-free period and the absence of clinical symptoms for at least 6 weeks.

## Results

The average follow-up period was about 27 months (5 months and 2 weeks to 14 years and 11 months). Altogether, 104 implants (91.2 %) fitted at our department were considered for this study, the drop-out rate being 8.8 %. The patient cohort comprised 30 men (29.7 %) and 71 women (70.3 %). Their average age at the time of surgery was 67 years (18–92 years), the average age of males being about 60 and of females about 70. Fifty-seven (50 %) of the implants had been fitted on the right side and 57 (50 %) on the left. In no cases was bilateral implantation carried out. The average BMI was about 27 ± 5 kg/m^2^ (16.9–43.8 kg/m^2^; *N =* 109).

Seven patients died within the first six months of non-infection-related causes. (Pulmonary embolism, tumor progression, etc.) These were not included in the further evaluation.

The indications were tumour (45 cases/39.5 %), TEP loosening (38/33.3 %), periprosthetic fracture (25/21.9 %), primary fracture (19/16.7 %), condition after TEP removal due to infection (11/9.6 %), pseudarthrosis (7/6.1 %) and recurrent dislocation of the TEP (1/0.9 %) (*N =* 146 with multiple indications). In 73 cases (64 %), previous operations had been carried out: primary THA implant (19 cases/26 %), THA replacement (15/20.5 %) and proximal femur replacement (8/11 %) as well as primary implantation of total knee arthroplasty (TKA) (12/16.4 %) and TKA replacement (2/2.7 %). There were another 8 cases (11 %) of osteosynthesis and 3 other operations (cement filling for osteomyelitis in childhood, corrective osteotomy and excochleation with filling).

Regarding localization, proximal femur replacement in 75 cases (65.8 %) was followed by distal femur replacement (28 cases/24.6 %), proximal tibia (4/3.5 %), total femur replacement (3/2.6 %) and diaphyseal replacement (3/2.6 %).

Endoprosthesis infections occurred in 19 cases (17,7 %), making it the second-most common complication after dislocation (20 cases/18.4 %). They comprised 3 early infections (within the first 6 weeks) and 16 late or delayed infections (between 9 weeks and 6 years and 7 months after implantation). Pathogens were identified in 14 cases; in 5 cases infection was clinically and paraclinically evident although the pathogens were not identified. The identified pathogens are listed in Table 1.

**Table 1:** Absolute and percentage frequency distribution of pathogen classes in the overall population (MDROs: multi-drug-resistant organisms; M/OR CNS: methicillin (oxacillin) resistant coagulase-negative staphylococci; MRSA: methicillin-resistant *Staphylococcus aureus*)

Coagulase-negative staphylococci	7	36.6 %
Staphylococcus epidermitis	5	26.3 %
Staphylococcus capitis	1	5.3 %
Staphylococcus warnei	1	5.3 %
thereof M/OR CNS	4	21.0 %
Staphylococcus aureus	3	15.8 %
thereof MRSA	1	5.3 %
Bacillus subtilis	1	5.3 %
Enterococcus faecalis	1	5.3 %
Pseudomonas aeruginosa	1	5.3 %
Escherichia coli	1	5.3 %
overall MDROs	5	26.0 %
no microbial detection	5	26.0 %

All infections were treated surgically. The only types of treatment used were debridement with lavage (2 cases), spacers followed by two or more replacements of the endoprosthesis (16 cases), removal without replacement of the prosthesis/ resection arthroplasty (3 cases), arthrodesis (3 cases) and amputation (2 cases). One patient (female) died from the consequences of infection. Seven of the 19 patients suffered reinfection of the modular endoprosthesis, making a reinfection rate of about 37 %.

There was no gender-based difference in infections (12 women and 7 men; insignificant (*p =* 0.464)).

Moreover, there were no significant differences between the ages of patients who did or did not develop an infection: 62 years (33–87 years) with infection and 67 without (18–92 years); insignificant (*p =* 0.729). The value of any findings would have been limited since according to the Kolmogorov-Smirnov test (*p =* 0.013) the age was not normally distributed.

Patients experiencing at least one infection had a BMI that on average was 5 points higher than patients with no infection (26 (19–37) patients with infection and 21 (14–44) patients without). However, this difference between the means is not significant either (*p =* 0.320) since the distribution of BMI in the cohort was approximately normal (Kolmogorov-Smirnov test, *p =* 0.630).

Patients with a tumour as the main indication for the implant of a modular endoprosthesis suffered significantly fewer infections than patients with other indications such as loosening, fractures, pseudarthrosis, etc. (tumours 8.9 % vs. 21.7 % other indications; *t*-test, *p =* 0.072).

In 73 patients, operations had been carried out beforehand on the relevant joint. After previous surgery, infections were about twice as likely to occur (previous surgery 20.5 % vs. no previous surgery 9.8 %, insignificant (*p =* 0.138)). Further classifying patients by types of previous operations would yield numbers of cases which are too low for statistical reliability.

Proximal femur replacement accounted for the largest share of all modular endoprostheses (65.8 %). Infections in connection with proximal femur replacement did not occur significantly more frequently than with other localizations (*p =* 0.427).

In the case of distal femur replacement, although infections did not take place more often than with other localizations (*p =* 0.119), the frequency of all complications considered together was somewhat higher than with other localizations.

## Discussion

**Fig. 2:**
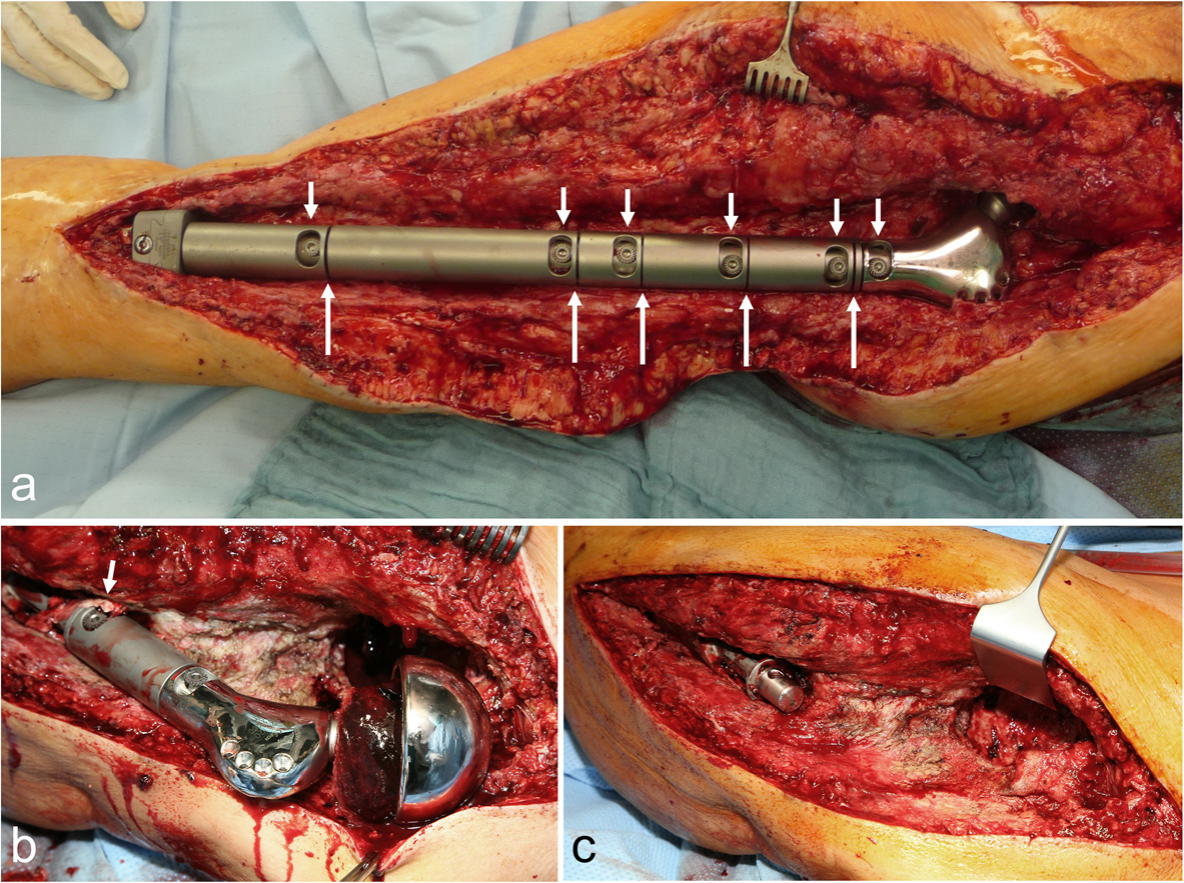
**a** Intraoperative situs with surgically treated complete femur replacement with multiple resulting grooves on the connection points (short arrows) and deep screw connections in the shaft (long arrows) **b** Intraoperative situs in connection with infected proximal femur replacement with inflammatory tissue in the screw holes (arrow) and on the head; **c** After debridement

Probably the biggest challenge when treating endoprosthesis complications is managing periprosthetic infections [7]. Particularly with large revision endoprostheses and thus also modular mega-endoprostheses, this area is of enormous importance. The infection rate in this study’s patient population was 17.7 %. Infection rates of between 4 % and 24 % are quoted in the literature, significantly higher than those encountered with primary implantations of the hip and knee (0.5–1.5 %) and also revision surgery (5 %) [10, 13–17]. Table 2 lists the current literature dealing with the occurrence of infections in connection with mega-endoprostheses. The reasons for this higher number of periprosthetic infections include the considerably larger foreign surface than on other endoprostheses. This large foreign surface as well as more extensive surgical wounds, lengthy operations and high blood loss increase the chance of infection [4]. Furthermore, the modular design with many dead spaces, gaps and grooves filling up with blood and wound fluid seems to be conducive to infections [7] (Fig. 2). The side effects of continuing oncological treatment may also constitute risk factors for infection [18]. For example, radiotherapy has been shown to be a risk factor, although this has not been proven for chemotherapy [19]. The connecting tubes used – additional non-perfused foreign bodies – may also promote and maintain infection [10].

**Table 2:** Percentage of infection frequency of modular mega-implants in the lower extremities in the literature

Author/ year	System	Number of patients	Locations	Follow-up time	Infections	Comments
Roberts et al. 1991[25]	STANMORE™	135	knee	not specified	6.8 %	
Unwin et al. 1996 [26]	STANMORE™	1001	hip, knee, femur shaft	46 months	24.6 %	
Mascard et al. 1998 [27]	GUEPAR™	90	knee	4.3 years (1–22 years)	13 %	
Ilyas et al. 2001 [28]	HMR- System	48	knee	5.6 years (2–10 years)	14.6 %	
Donati et al. 2001 [15]	KMFTR-System	34	knee	10 years	4 %	
Mittermayer et al. 2001 [16]	KMFTR-System	100	hip, knee, femur shaft	127.5 months	9.7 %	51 patients died and 41 patients analysed
Ilyas et al. 2002 [14]	HMR- System	15	knee	6.7 years (3–10 years)	13.3 %	
Griffin et al. 2005 [29]	several	99	hip, knee, femur shaft	6 years (3.2–158.9 months)	10.1 %	
Heisel et al. 2006 [17]	MUTARS	50	hip, knee, femur shaft	46 monts (2–7 years)	12 %	
Gosheger et al. 2006 [13]	MUTARS	250	hip, knee, femur shaft	not specified	12 %	
Gerdesmeyer et al. 2006 [30]	MML-System	70	hip, knee, femur shaft	7 years +/− 28 months	none	drop-out 46 %
Gradinger and Gollwitzer 2006 [12]	MML-System	89	hip, knee, femur shaft	not specified	3.3 %	
Hardes et al. 2009 [31]	MUTARS	28	hip, knee	not specified	7.1 %	without tumor
Chandrasekar 2009 [32]	STANMORE™	100	hip	24.6 months (0–60 months)	4 %	
Kinkel et al. 2010 [33]	MUTARS	77	hip, knee, femur shaft	not specified	11.7 %	60 % complications
von Salis-Soglio et al. 2010 [10]	MML-System	572	hip, knee, femur shaft	not specified	10.5 %	
Ruggeri et al. 2010 [34]	KMFTR, HMRS, GMRS	28	complete femur	8 months (1 month −17 years)	7.1 %	
Winkelmann et al. 2010 [35]	MUTARS	41	hip, knee, femur shaft	45 months	19.5 %	
Pala et al. 2015 [36]	GMRS	247	knee	4 years (2–8 years)	9.3 %	
Capanna et al. 2015 [37]	several	278	hip, knee	more than 2 years	8.3 %	
Sevelda et al. 2015 [38]	several	50	complete femur	57 months (1–280 months)	12 %

Another major factor is the patient’s history regarding previous infections and operations. It was shown in our study that when tumours were the main diagnosis for the implant of a modular endoprosthesis, this resulted in significantly fewer infections than the other indications. In particular, foregoing infections play an important role in the recurrence of a periprosthetic infection [7]. Ascherl et al. report that a significantly higher rate of infection is to be expected in connection with revisions after periprosthetic infection compared to primarily aseptic replacement involving mega-endoprostheses [7]. Ahrens et al. found that reinfection was suffered by 43 % of patients who had recovered from infection [20]. This was borne out by the high reinfection rate in our own study, in which reinfection accounted for 7 of the 19 infections (37 %). High priority must be attached to preventing infection [4].

Infection must be correctly diagnosed and classified for appropriate therapy [21]. For infections of mega-prostheses, the classification system devised by Tsukayama et al. is recommended [7, 22]:

I. Positive culture of intraoperative samples with no previous indication of infection
II. Early infection: Onset of symptoms within 1 month
III. Chronic infection: Symptoms after 1 month
IV. (Acute) Hematogenous infection (2 years after surgery)

Using this system is logical since it forms the basis for the therapeutic approach. Positive intraoperative detection of pathogens with no other clinical or paraclinical evidence of infection (Tsukayama Type I) could in particular be legally problematical. In uncertain cases, an infection could be either simple contamination or a previously asymptomatic early infection. Therefore, taking multiple intraoperative samples (at least 5) is important: if at least 3 show the same pathogen, infection seems likely and should be treated as such [7, 21]. An unusual spectrum of pathogens (enterobacteria, etc.) is unlikely to be contamination and should be critically examined. In these cases, taking more samples by puncture is advisable [21]. In cases of doubt, taking a consistent approach and treating such findings as an early infection always appears to be the preferable response, even if this remains an individual decision taken on a case-by-case basis.

In the event of early infection (Tsukayama Type II), open surgical revision should always be performed; antibiotic treatment or arthroscopic flushing is not effective by itself. The wound should be completely reopened and multiple samples taken in all layers followed by consistent debridement and lavage. The complete replacement of all movable endoprosthesis elements (PE bearings, heads, etc.) as well as of non-cemented, screwed or jammed modules and any connecting tubes used is essential. In this regard, it is vital to make sure preoperatively that the existing implants are complete and to check for possible alternatives (perhaps by consulting the respective manufacturer). In addition, local and systemic antibiosis should be used, ideally in connection with known pathogen patterns. The possible adoption of a serial approach must be decided on a case-by-case basis, since the functional outcome deteriorates with each further revision in conjunction with often limited muscle and soft tissue (sufficient soft tissue coverage is essential for infection control) [7]. Generally, however, the focus should always be on consistently curing the infection.

**Fig. 3:**
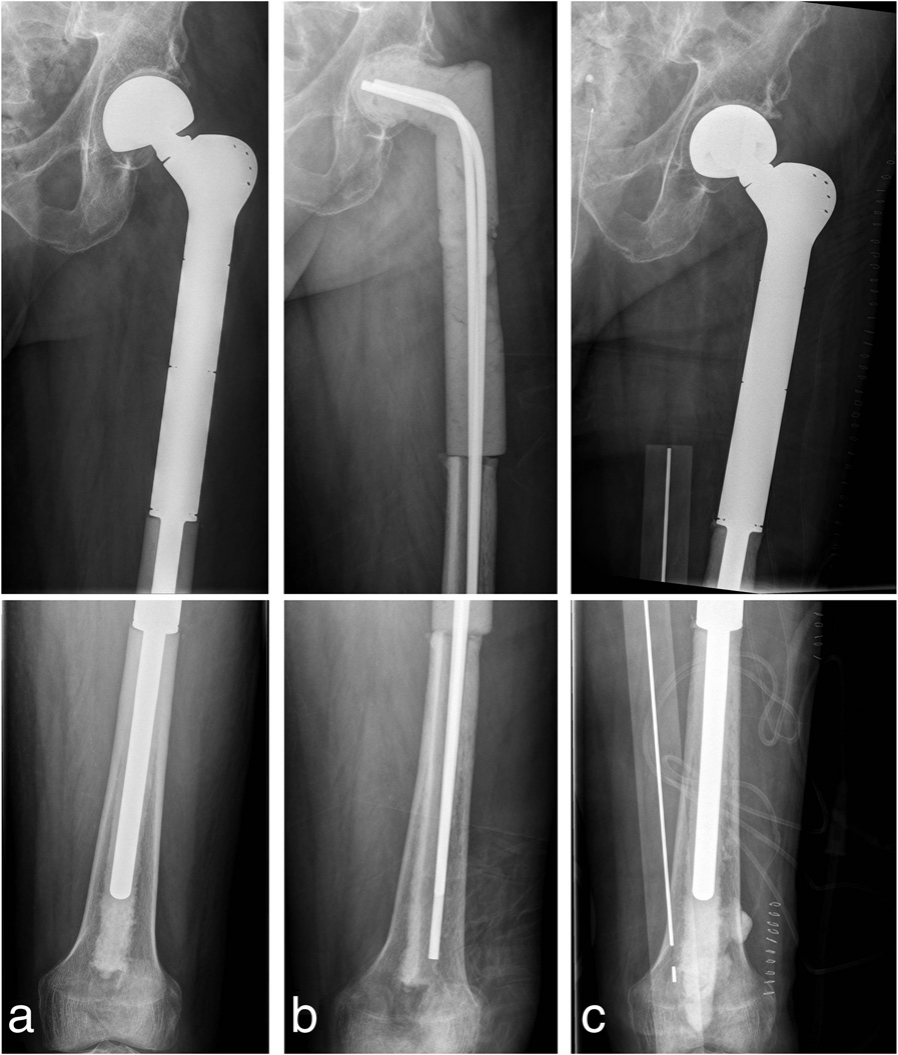
**a** Anterior-posterior X-ray of infected femur replacement (chronic infection); **b** After removal and with inserted cement spacer; **c** After reimplantation of a proximal femur replacement

**Fig. 4:**
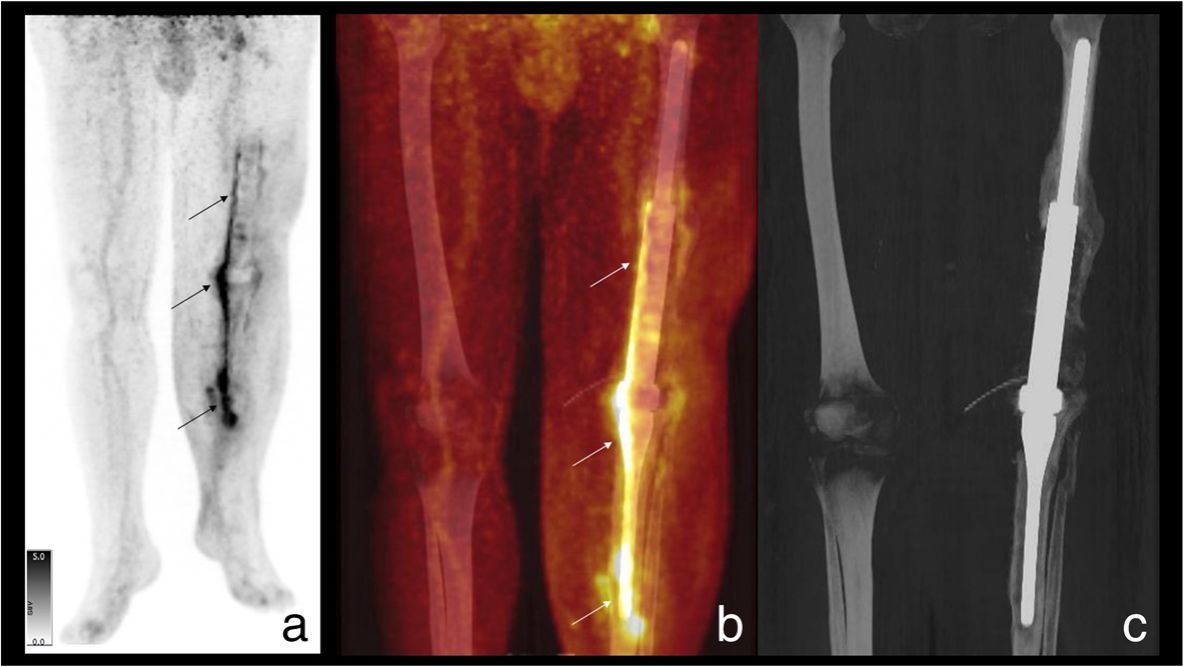
[18 F]FDG PET/CT: **a** Maximum intensity projection (MIP) of [18 F]FDG-PET; **b** MIP of [18 F]FDG PET/CT fusion and **c** MIP of CT (bony window): clearly increased glucose consumption along the medial side of the arthrodesis (arrows) and punctum maximum at the distal end with extension into the soft tissue as evidence of a prosthesis infection

**Fig. 5:**
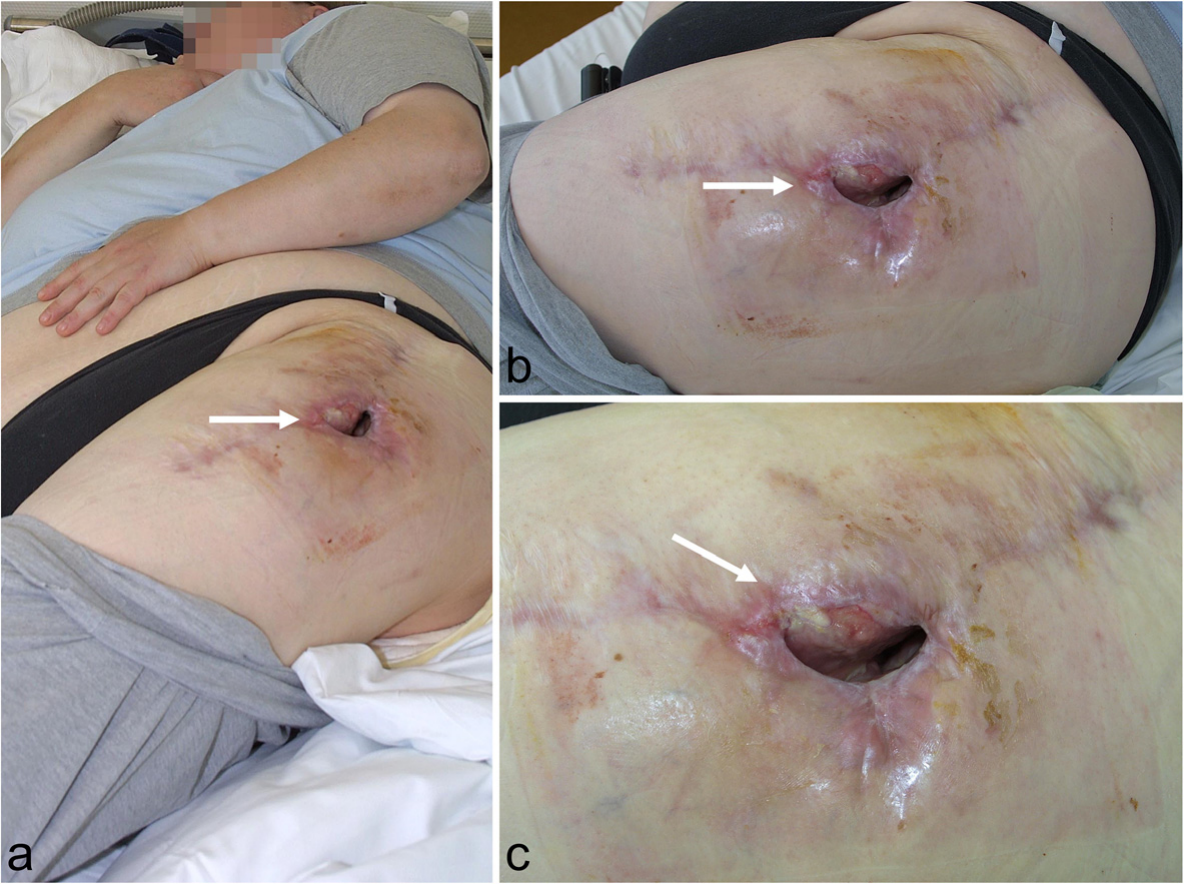
Obese 45-year-old patient with chronic fistula of the left hip in connection with inserted proximal femur replacement after multiple futile infection eradiation: **a** overview; **b** and **c** detailed views

In chronic infection 4–6 weeks after implantation (Tsukayama Type III), a different procedure is required since the initial colonization of the implant and the possible formation of a biofilm must be assumed [21]. The endoprosthesis cannot usually be preserved in such cases. A one-stage replacement as in primary periprosthetic infection is often ineffective. Instead, in our view, a multi-stage approach should always be taken involving the complete removal of all implant parts, the radical resection of infected tissue with debridement and lavage, and local and systemic antibiosis. Cement spacers, provisional prostheses or external fixators and extensions can be used for temporary stabilization depending on the location and extent of the defect, although with defects often being extensive, such interim treatment is frequently complicated and functionally unsatisfactory for the patient. Once again, a serial approach is often necessary. Once the infection has been cured, in ideal cases reimplantation is possible (Fig. 3). However, even larger defects often arise due to surgical debridement, requiring extended modules. One possible solution is the use of arthrodesis modules, especially in the knee area. Even so, they often have to be longitudinally cemented in the tibia and femur, and in addition to their limited functional results, salvage procedures upon reinfection are barely possible (Fig. 4). Stabilizing the fistula is usually only briefly effective (if at all) due to the multi-cavity situses as well as the distal and proximal spread of infection (Fig. 5). In 3 cases, the endoprosthesis was removed owing to a serious infection and resultant spacer arthrodesis, leading in 1 case to amputation and another case eventually to death. The use of arthrodesis after the infection of a modular endoprosthesis is therefore only partly suitable as palliation to avoid amputation [7]. In the event of infection, the salvage possibilities are limited and extremely difficult. This underlines the risk and serious consequences of infection in connection with a lying modular endoprosthesis. Alongside tumour progression, infection in connection with an implanted modular endoprosthesis is one of the most common reasons for the amputation of the affected limb [18].

According to the literature, ‘hematogenous’ early infections (Tsukayama Type IV) can in some cases with sudden fulminant symptoms also be treated in isolation antiobiotically. However, this approach has never been successful at our department and therefore appears ineffective in connection with large implants. Once again, as with chronic infection, a surgical procedure (see above) is advisable. In individual cases, a single attempt can be made to preserve the implant as with early infection, but should be discussed in great detail.

In some works, titanium implants [20] and silver-coated implants [23] are reported to result in a lower rate of infection than cobalt-chromium based alloys. The surface of silver-coated endoprostheses has an anti-infective effect, as already demonstrated in animal experiments [23] and in vivo studies [24]. Silver’s pharmacological safety has also been demonstrated, for although silver ions were released, no side effects were observed as a result [20, 24]. Moreover, stocking-shaped fabrics (e.g. polyethylene terephthalate) have been described which, when introduced above the implants, are supposed to promote healing and seroma formation [7]. However, this method cannot solve the complex problems entirely.

Another significant problem is the expected pathogen pattern. Our own work revealed a similar pathogen spectrum as in primary endoprosthesis infections with the predominance of *Staphylococcus aureus* and coagulase-negative staphylococci (Table 1). Strikingly, about 25 % of the pathogens showed a multi-resistant antibiotic spectrum. Back in 2010, Ascherl described the further progress of the multi-resistant pathogens MRSA (methicillin-resistant Staphylococcus aureus) and MRSE (methicillin-resistant Staphylococcus epidermidis) in modular TEP infections [7]. In particular due to the formation of biofilms, the collection of colonies, and low vulnerability to sometimes only bacteriostatic drugs (linezolid), an endoprosthesis-conserving approach in connection with MDROs (multi-drug resistant organism) is only successful in isolated cases and should not be attempted. Parvizi reports a 40 % reinfection rate for infections of conventional endoprostheses by multi-resistant staphylococci, which means the rate in connection with modular prostheses is probably much higher. The treatment of MDROs is and remains the main challenge in the management of endoprosthesis infections.

## Conclusion

The 18 % rate of infection of modular endoprostheses (4–36 % in the literature) is significantly higher than that of primary endoprostheses and the most frequent complication alongside the dislocation of the proximal femur replacement. Infection control is challenging due to limited salvage procedures, the high recurrence rates and patients’ frequent multi-morbidity. For therapy, Tsukayama’s classification system should be applied, and a consistent, radical surgical procedure is essential. Even with an adequate treatment concept, the recurrence rate is very high at 36–43 %. Unfortunately, unsatisfactory functional results often result, with amputation frequently being the last resort.

Therefore, indications for implantation must be carefully considered and critically discussed, especially regarding multi-morbid patients with previous joint infections.

## Limitations

The retrospective design, the inhomogeneity of the patient population, and the relatively short follow-up period are limitations of the present study. However, this applies to the majority of studies of infected modular mega-endoprostheses.
